# A risk index for COVID-19 severity is associated with COVID-19 mortality in New York City

**DOI:** 10.1186/s12889-021-11498-x

**Published:** 2021-07-24

**Authors:** Wil Lieberman-Cribbin, Naomi Alpert, Raja Flores, Emanuela Taioli

**Affiliations:** 1 Institute for Translational Epidemiology and Department of Population Health Science and Policy, Icahn School of Medicine at Mount Sinai, One Gustave L. Levy Place, Box 1133, New York, NY 10029 USA; 2 Department of Thoracic Surgery, Icahn School of Medicine at Mount Sinai, One Gustave L. Levy Place, Box 1023, New York, NY 10029 USA

**Keywords:** Mortality, Coronavirus, Comorbidities

## Abstract

**Background:**

New York City (NYC) was the epicenter of the COVID-19 pandemic, and is home to underserved populations with higher prevalence of chronic conditions that put them in danger of more serious infection. Little is known about how the presence of chronic risk factors correlates with mortality at the population level. Here we determine the relationship between these factors and COVD-19 mortality in NYC.

**Methods:**

A cross-sectional study of mortality data obtained from the NYC Coronavirus data repository (03/02/2020–07/06/2020) and the prevalence of neighborhood-level risk factors for COVID-19 severity was performed. A risk index was created based on the CDC criteria for risk of severe illness and complications from COVID-19, and stepwise linear regression was implemented to predict the COVID-19 mortality rate across NYC zip code tabulation areas (ZCTAs) utilizing the risk index, median age, socioeconomic status index, and the racial and Hispanic composition at the ZCTA-level as predictors.

**Results:**

The COVID-19 death rate per 100,000 persons significantly decreased with the increasing proportion of white residents (β_adj_ = − 0.91, SE = 0.31, *p* = 0.0037), while the increasing proportion of Hispanic residents (β_adj_ = 0.90, SE = 0.38, *p* = 0.0200), median age (β_adj_ = 3.45, SE = 1.74, *p* = 0.0489), and COVID-19 severity risk index (β_adj_ = 5.84, SE = 0.82, *p* <  0.001) were statistically significantly positively associated with death rates.

**Conclusions:**

Disparities in COVID-19 mortality exist across NYC and these vulnerable areas require increased attention, including repeated and widespread testing, to minimize the threat of serious illness and mortality.

**Supplementary Information:**

The online version contains supplementary material available at 10.1186/s12889-021-11498-x.

## Background

New York City was once considered the epicenter of the coronavirus disease (COVID-19) epidemic in the United States. From the first reported case on March 1, 2020, to April 2020, nearly 600 people were dying each day, new daily cases numbered in the thousands, and new hospitalizations were roughly 1500 per day [1]. The mortality count peaked on April 7, where there were 598 reported deaths, 6042 cases and 1578 people hospitalized. Reflecting on this time has highlighted racial and socioeconomic disparities in COVID-19 testing patterns that are indicative of gaps in initial COVID-19 response policy [2–5]. Analysis of COVID-19 case identification and mortality rates in New York City has also underscored variations in the amount of COVID-19 testing performed over time, delays in receiving testing results, and lags in the reporting of death data [6, 7]. To provide a more complete picture of mortality data, the New York City Department of Health and Mental Hygiene COVID-19 Response Team issued a publication on the topic and reported that over 5000 excess deaths occurred from the COVID-19 pandemic from March 11th through May 2nd [8]. Within NYC, cumulative mortality rates are highest in Hispanic/Latino and African American/Black populations, as well as older age groups [1].

Clinical studies across the US and NYC have shown that people with underlying health conditions and risk factors, including diabetes, chronic lung disease, and cardiovascular disease, are at higher risk for severe outcomes and mortality from COVID-19 [1, 9–11]. However, little is known about how the presence of these chronic risk factors correlates with mortality at the population level [12–14]. Such information is key to developing effective public health interventions to mitigate COVID-19 spread and adverse outcomes, and population studies can guide policy at the community-level. In addition, population level studies are enriched with minorities and the whole spectrum of socioeconomic status, thus reflecting reality more closely. A study of US data looked at the prevalence of risk factors for COVID-19 severity according to CDC guidelines, and asserted that people living in low-income households and those who are Black or American Indian are more likely to have chronic conditions that place them in a higher risk category for COVID-19 complications [12]. The study however did not correlate this high-risk profile with mortality data. Within NYC, the distribution of behavioral risk factors and chronic conditions is highly variable from neighborhood to neighborhood; higher obesity rates in Brooklyn and the Bronx have been posited as being partially responsible for the increased COVID-19 mortality in these areas [15]. A separate study reported older age and severe obesity as risk factors for COVID-19 mortality and worse in-hospital outcomes in a Bronx cohort [16]. However, the research published so far only partially tackles a more complex reality when studying COVID-19. Minorities and underserved populations have higher prevalence of chronic conditions that put them in danger of a more serious infection; at the same time, they also have higher risk of exposure to the virus, as they frequently live in neighborhoods with higher population density and proportions of essential workers, with fewer opportunities to exercise social distancing [17–19]. Here we study risk factors associated with COVD-19 mortality at the neighborhood level in NYC. We hypothesized that areas with the highest prevalence of risk factors, minority residents and lower SES experienced the highest mortality in NYC.

## Methods

### Risk index

Risk factors were identified based on the CDC criteria for risk of severe illness and complications from COVID-19 [20]. Census tract level crude prevalence (%) of diabetes, asthma, chronic obstructive pulmonary disease (COPD)/emphysema/chronic bronchitis, cancer (besides skin), angina/coronary heart disease, chronic kidney disease, obesity, and hypertension were extracted from the 500 Cities Project [21], which sourced this data from the 2017 Behavioral Risk Factor Surveillance System (BRFSS) [22]. The New York City Department of Health and Mental Hygiene online tool EpiQuery [23] was used to extract United Hospital Fund (UHF) neighborhood level information on prevalence of hepatitis B and C from 2017 Communicable Disease Surveillance Data. ZIP code level rates, based on the population of each zip code, of alcohol related hospitalizations in 2017 were extracted from New York State’s hospital discharge database [24] based on the following ICD-10 diagnosis codes: F10, G62.1, I42.6, K29.20, K29.21, K70.10, K70.11, K70.30, K70.2, K70.31, K70.4, K70.9, K73.0-K73.9, K74.0, K74.2, K76.9, K76.6, K86.0, K86.1 (diagnoses of alcohol-related morbidity), R78.0, and T51 (alcohol poisoning). Hepatitis B, C, and alcohol related hospitalizations were used as proxies for liver disease. Epiquery was also used to assess 2015 birth rates by UHF, as a proxy for pregnancy, while heart attack hospitalizations by UHF in 2016 were extracted from New York City Environment and Health Data Portal [25]. The percent of the population 65 years and older was extracted from American Community Survey 2018 5-year estimates. The median age, proportion of white, and proportion of Hispanic residents were also extracted from 2018 census data. Median age was extracted despite the inclusion of age as a risk factor in order to adjust for different age distributions across NYC, as the prevalence of risk factors were not age adjusted. Data for all indicators were converted to ZIP code, using crosswalks provided by the census [26] and the New York City Department of Health (NYC DOH)[27]. A summary table of the risk factor measures and their citywide prevalence is included in Supplementary Table 1.

Each risk factor was then divided into quartiles and assigned a score of 1 to 4, with the lowest quartile (score 1) representing areas with the lowest severe COVID-19 risk associated with a given variable, and the highest quartile (score 4) representing the highest risk. Risk quartiles for Hepatitis B, C, and alcohol related hospitalizations were summed to create one variable capturing liver disease. These scores were then summed across all risk factors into a single score for each ZCTA, with higher values corresponding to higher prevalence of risk factors. NYC ZCTAs were classified into quartiles of this risk score.

### Socioeconomic status index

Zip Code Tabulation Area (ZCTA) level data on median household income in the past 12 months (Table B19013), median gross rent (B25064), percent living below 150% of the poverty line (Table C17002), education (B15002), percent working class (C24010), percent unemployed (B23025), and >  1 occupants per room (B25014) was downloaded from 2018 American Community Survey (ACS) 5-year estimates [28]. These variables were combined into an index of socioeconomic status using principal component analysis (PCA), as previously described [2]. A linear combination was then created to obtain an SES index score assigned to each ZCTA. A lower SES score corresponds to a lower SES, while a higher score represents higher SES.

### COVID-19 mortality by zip code tabulation area

The COVID-19 related death count and the COVID-19 death rate were downloaded from the NYC Coronavirus (COVID-19) data repository [1] hosted by the NYCDOH from March 2nd (when the repository began publishing data) to July 6th, when Phase 3 of reopening started in NYC. The death rate, the risk sum, each component of the risk index, median age, SES index, and racial and Hispanic composition by ZCTA were mapped in ArcGIS v10.7.1. Deaths counts reflect people’s ZCTA of residence at the time of reporting, not the location of death.

### Statistical analysis

The geographic unit of analysis was the ZCTA, where each ZCTA has a risk index score, SES index score, and mortality information. Wilcoxon rank-sum tests were performed to assess differences in COVID-19 death rate, risk index components, racial and ethnic composition, and median age according to risk index quartiles. Spearman correlations were performed to assess the association between the COVID-19 death rate per 100,000 residents, the risk index, median age, SES index, and the racial and Hispanic composition of the ZCTA. Stepwise linear regression was performed to predict the COVID-19 death rate per 100,000 residents through July 6th utilizing the risk index, median age, socioeconomic status index, and the racial and Hispanic composition at the ZCTA level as predictors. Variables that were significant at the α = 0.25 level were considered for entry into the multivariable model and those that were significant at the α = 0.15 level after adjustment remained in the final model. All analyses were performed in SAS v9.4.

## Results

### Risk index

Each component of the risk index, as well as the proportion of white and Hispanic residents, median age, SES index and COVID-19 death rate were analyzed according to quartiles of the risk index (Table 1). Across risk index quartiles representing increasing risk, the COVID-19 death rate per 100,000 (*p* <  0.0001), asthma prevalence (*p* <  0.0001), kidney disease prevalence (*p* <  0.0001), hypertension prevalence (*p* <  0.0001), heart disease prevalence (*p* <  0.0001) obesity prevalence (*p* <  0.0001), COPD prevalence (*p* <  0.0001), diabetes prevalence (*p* <  0.0001), Hepatitis C (*p* <  0.0001), Hepatitis B (*p* = 0.0055), proportion of residents greater than 65 years old (*p* = 0.0378), birth rate (*p* = 0.0325), alcohol hospitalizations (*p* = 0.0005), and the proportion of Hispanic residents (*p* = 0.0004) increased, while the proportion white residents (*p* <  0.0001) and the SES index (*p* <  0.0001) decreased. All-cancer incidence rates (*p* = 0.1333) and median age (*p* = 0.1326) were similar across risk sum quartiles.

**Table 1:** Description of COVID-19 death rate per 100,000 residents, risk index components, and socioeconomic status index according to quartiles of the risk index

Variable	Quartile 1 (lowest risk score; 21.46–40.26) mean (SD)	Quartile 2 (40.27–48.15) mean (SD)	Quartile 3 (48.16–55.01) mean (SD)	Quartile 4 (highest risk score; 55.02–73.84) mean (SD)	*p*-value
COVID-19 death rate per 100,000	109.72 (68.83)	192.78 (72.51)	251.98 (97.16)	284.16 (112.68)	< 0.0001
Median age (years)	35.4 (3.31)	37.64 (3.81)	36.71 (4.26)	36.73 (6.23)	0.1326
Proportion white residents (%)	66.68 (15.40)	45.89 (21.26)	36.78 (23.53)	32.21 (26.81)	< 0.0001
Proportion Hispanic residents (%)	17.12 (12.400	29.13 (18.53)	25.67 (18.82)	34.52 (22.98)	0.0004
Obesity prevalence (%)	17.87 (5.01)	22.31 (4.24)	26.53 (4.69)	29.72 (5.98)	< 0.0001
Kidney disease prevalence (%)	2.17 (0.49)	2.84 (0.27)	3.20 (0.26)	3.67 (0.48)	< 0.0001
Hypertension prevalence (%)	20.36 (4.00)	26.43 (2.15)	30.37 (3.13)	33.60 (3.41)	< 0.0001
Heart Disease prevalence (%)	3.75 (1.01)	5.25 (0.42)	5.76 (0.59)	6.49 (0.81)	< 0.0001
Diabetes prevalence (%)	6.87 (2.27)	10.48 (1.58)	11.78 (1.40)	13.13 (2.17)	< 0.0001
COPD prevalence (%)	3.78 (1.11)	5.17 (0.62)	6.04 (0.72)	6.75 (0.90)	< 0.0001
Cancer (except skin) prevalence (%)	5.02 (1.14)	5.49 (1.03)	5.55 (0.94	5.79 (1.53)	0.1333
Asthma prevalence (%)	8.46 (0.74)	8.92 (0.95)	10.11 (1.34)	10.80 (1.26)	< 0.0001
Alcohol Hospitalizations prevalence (%)	1.71 (1.02)	1.36 (0.56)	1.51 (0.77)	2.26 (1.20)	0.0005
Birth rate (%)	1.25 (0.37)	1.16 (0.32)	1.26 (0.32)	1.30 (0.23)	0.0325
Hepatitis C prevalence (%)	0.05 (0.02)	0.05 (0.01)	0.05 (0.01)	0.08 (0.03)	< 0.0001
Hepatitis B prevalence (%)	0.06 (0.07)	0.09 (0.09)	0.08 (0.07)	0.07 (0.04)	< 0.0001
Proportion ≥ 65 years old (%)	12.3 (4.7)	14.88 (4.09)	14.51 (3.72)	15.73 (5.95)	0.0378
Socioeconomic status index	33.87 (21.38)	12.03 (17.72)	−1.20 (18.74)	−16.37 (22.08)	< 0.0001

The racial and Hispanic composition of each ZCTA were obtained from the 2018 American Community Survey 5-year estimates. Unless otherwise noted, all variables are reported per hundreds of residents. The socioeconomic status index was constructed from principal component analysis of 2018 American Community Survey estimates of median household income in the past 12 months, median gross rent, percent living below 150% of the poverty line, education, percent working class, percent unemployed, > 1 occupants per room. The education index was calculated on the population ≥ 25 years and is a weighted combination of the percent high school graduate, high school only, and more than high school, with a greater value indicating higher educational attainment

Correlations among the COVID-19 death rate, risk index, proportion White residents, proportion Hispanic residents, the median age, and the SES index (Table 2) indicated that the COVID-19 death rate was significantly positively correlated with the risk index (*ρ* = 0.67, *p* <  0.0001), increasing proportion of Hispanic residents (*ρ* = 0.42, *p* <  0.0001), and significantly inversely correlated with the proportion of white residents (*ρ* = − 0.56, *p* <  0.0001) and the SES index (*ρ* = − 0.63, *p* <  0.0001). The risk index was significantly positively correlated with the increasing proportion of Hispanic residents (*ρ* = 0.25, *p* = 0.0012), and significantly inversely correlated with the proportion white residents (*ρ* = − 0.48, *p* <  0.0001) and the SES index (*ρ* = − 0.67, *p* <  0.0001). The correlation between the risk index components and the SES index is reported in Supplementary Table 2.

**Table 2:** Correlations among predictors and COVID-19 death rate per 100,000 residents

	Statistic	Death Rate Up to July 6	Risk Index	White Proportion (%)	Hispanic Proportion (%)	Median Age (years)	SES Index
Death Rate up to July 6	ρ	–	0.67	−0.56	0.42	0.03	−0.63
	p-value	–	<0.0001	<0.0001	<0.0001	0.6441	<0.0001
Risk Index	ρ	–	–	− 0.48	0.25	0.08	−0.67
	p-value	–	–	<0.0001	0.0012	0.27	<0.0001
White Proportion (%)	ρ	–	–	–	−0.40	0.32	0.74
	p-value	–	–	–	<0.0001	<0.0001	<0.0001
Hispanic Proportion (%)	ρ	–	–	–	–	−0.39	−0.69
	p-value	–	–	–	–	<0.0001	<0.0001
Median Age (years)	ρ	–	–	–	–	–	0.37
	p-value	–	–	–	–	–	<0.0001

Note: Spearman correlations were performed

The socioeconomic status index was constructed from principal component analysis of 2018 American Community Survey estimates of median household income in the past 12 months, median gross rent, percent living below 150% of the poverty line, education, percent working class, percent unemployed, > 1 occupants per room. The education index was calculated on the population ≥ 25 years and is a weighted combination of the percent high school graduate, high school only, and more than high school, with a greater value indicating higher educational attainment

### Geography of COVID-19 death rates

**Fig. 1:**
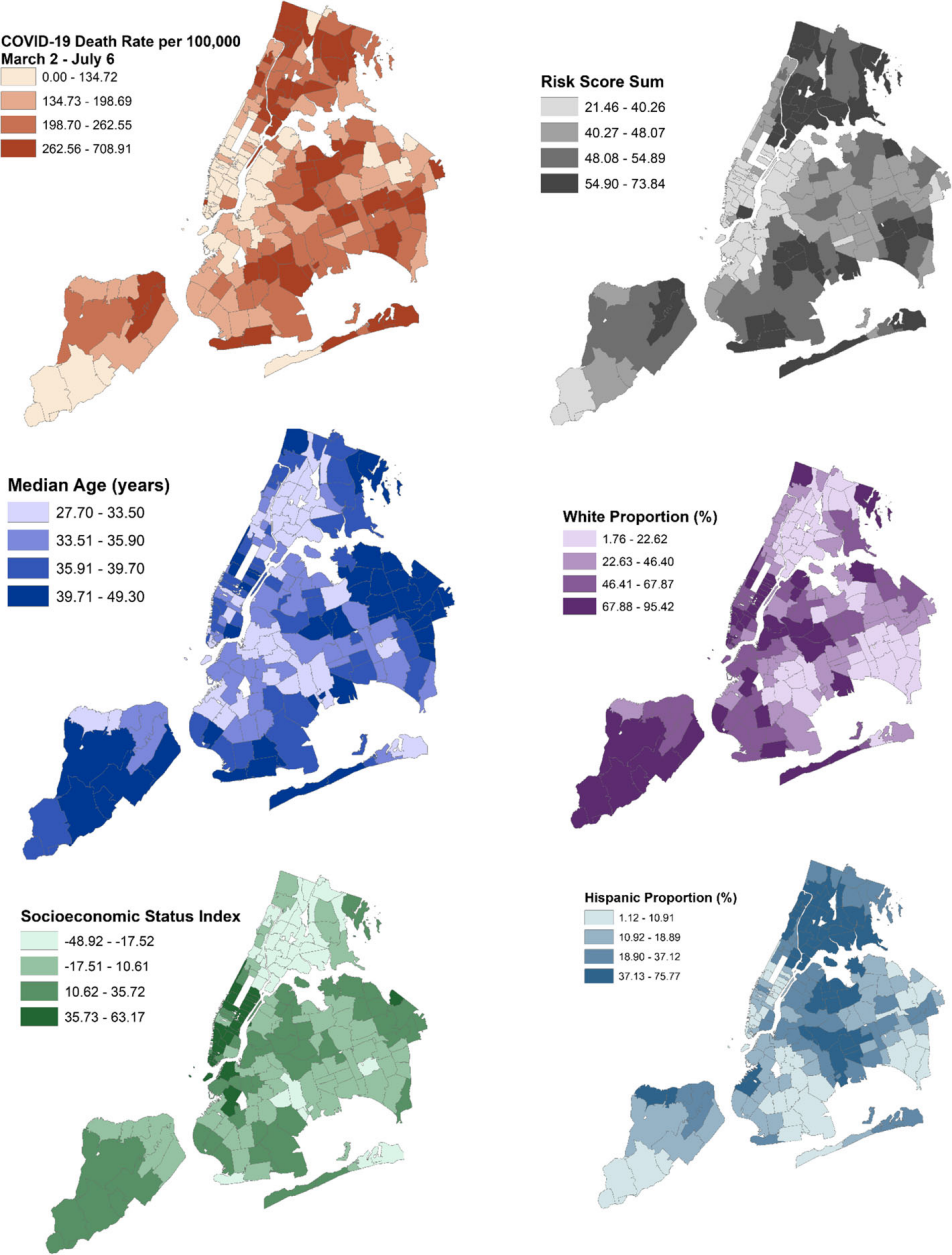
Distribution of the COVID-19 death rate per 100,000 residents from March 2nd to July 6th (top-left), the risk index (top-right), the median age (middle-left), the proportion white residents (middle-right), the socioeconomic status index (bottom-left) and the Hispanic composition (bottom-right) across New York City Zip Code Tabulation Areas. The risk index is a sum score of quartiles of asthma prevalence + cancer prevalence (excluding skin cancer) + COPD prevalence + diabetes prevalence + heart disease prevalence + hypertension prevalence + kidney disease prevalence + obesity prevalence + Heart attack prevalence + proportion of the population aged ≥65 years + birthrate prevalence and liver disease (measured by summing quartiles of Hepatitis B prevalence, Hepatitis C prevalence and alcohol hospitalizations prevalence). The socioeconomic status index was constructed from principal component analysis of 2018 American Community Survey estimates of median household income in the past 12 months, median gross rent, percent living below 150% of the poverty line, education, percent working class, percent unemployed, > 1 occupants per room. The education index was calculated on the population ≥ 25 years and is a weighted combination of the percent high school graduate, high school only, and more than high school, with a greater value indicating higher educational attainment. This figure was created using our licensed copy of ArcGIS (version 10.7.1; ESRI, Redlands, CA)

There are 177 ZCTAs in NYC reporting COVID-19 death rate data and 18,851 cumulative deaths in NYC as of July 6, 2020. The distribution of the COVID-19 death rate were mapped according to each ZCTA, as well as the risk index, the median age, the SES index, and the racial and Hispanic proportion (Fig. 1). Death rates per 100,000 residents were highest in the most Northern portion of NYC encompassing the Bronx, and were lowest in Manhattan. There was heterogeneity among ZCTAs in other NYC boroughs, including in Staten Island, which has low death rates in the southwest but high death rates in the northeast portion of the borough. Likewise areas of central Queens and eastern Brooklyn have high death rates, while death rates decrease in areas closer to Manhattan in these boroughs. Areas with high death rates have noticeable overlap with the risk score and inverse correlations with socioeconomic index, such as the Bronx. However, areas of central Queens with high death rates have moderate risk and socioeconomic scores, illustrating the complex interplay between risk factors and mortality.

### Predicting COVID-19 death rates

In the final multivariable model, the COVID-19 death rate per 100,000 persons significantly decreased with the increasing proportion of white residents in the ZCTA (β_adj_ = − 0.91, SE = 0.31, *p* = 0.0037), while increasing proportion of Hispanic residents (β_adj_ = 0.90, SE = 0.38, *p* = 0.0200), median age (β_adj_ = 3.45, SE = 1.74, *p* = 0.0489), and risk index (β_adj_ = 5.84, SE = 0.82, *p* <  0.001) were statistically significantly positively associated with death rates (Table 3).

**Table 3:** Predictors of cumulative COVID-19 death rate per 100,000 residents

	β_adj_ (SE)	*P* value
Intercept	− 176.12 (61.35)	0.005
White residents (%)	−0.91 (0.31)	0.004
Hispanic Composition (%)	0.90 (0.38)	0.02
Median Age (years)	3.45 (1.74)	0.049
Risk Index	5.84 (0.82)	< 0.0001

^a^ Stepwise linear regression was performed, and originally includes white residents, Hispanic composition, median age, risk index, and socioeconomic status index. SES index was removed from the model

Models were adjusted for all variables shown. SE: standard error. The risk index is a sum score of quartiles of asthma prevalence + cancer prevalence (excluding skin cancer) + COPD prevalence + diabetes prevalence + heart disease prevalence + hypertension prevalence + kidney disease prevalence + obesity prevalence + Heart attack prevalence + proportion of the population aged ≥65 years + birthrate prevalence and liver disease (measured by summing quartiles of Hepatitis B prevalence, Hepatitis C prevalence and alcohol hospitalizations prevalence)

## Discussion

This is the first analysis to incorporate CDC-defined risk factors for severe COVID-19 illness, NYC mortality data, racial and ethnic composition, and SES to predict COVID-19 death rates across NYC at the neighborhood level. Here we identify that areas with fewer white residents, more Hispanic residents, older residents, and with higher prevalence of a greater number of risk factors had increased COVID-19 mortality. These findings expand on existing literature that has partially addressed the potential risk of severe COVID-19 illness deriving from the complex intersection of chronic risk factors, SES and race [12] and can be useful in plans to address and reduce excess mortality in NYC^5^. In the present analysis, the COVID-19 death rate was highest in areas with the highest prevalance of risk factors, including hypertension, obesity, diabetes, asthma, and older age. Within NYC, these areas of greatest risk correspond to the Bronx, southeastern and southern Brooklyn, and parts of southern Queens; areas that are also of lower socioeconomic status with larger resident minority populations. Past neighboorhood analysis of NYC communities has reported that these areas have limited capacity to social distance, as measured by NYC subway data, and therefore have higher infection risk [29]. It is possible that these areas contain a large proportion of essential workers, such as healthcare, transportation and public-service workers, who are unable to work from home. It has been cited that social distancting, including working from home, is a privilege [18], and this is not a reality in communities characterized by higher proportions of comorbidites, risk factors for COVID-19, and adverse social determinants of health, including potential overcrowding and food insecurity. Further disparities in SES and healthcare access would only exacerabate adverse circumstances in these communities and contribute to increased risk of infection and mortality.

It has been noted that blanket public health recommendations in regards to social distancing for COVID-19 do not take local contexts and populations into account [17], and may be applied less effectively in socioeconomically disadvatanged and minorty communities due to several factors, including overcrowded households and higher proportions of essential workers. Instead, understanding local contexts is central in creating effective public health solutions. One avenue to begin to rectify disparities is to perform increased testing in these communities [2], and more specifically in sub-groups at higher risk of serious COVID-19 complications. However, there remains a gap in appropriate testing and attention given to these vulnerable communites [2]. Here, we emphasize that many of the same communities that are more suceptible to increased COVID-19 spread also face higher risks of severe illness and mortality if infected. The data point to the need for more aggressive actions in preventing COVID-19 spread and detecting early infections in specific areas of NYC.

One limitation of this analysis is that the data used are aggregate ZCTA-level data, which limits our ability to draw individual-level conclusions. For instance, we cannot comment on the interaction between being non-white and having chronic risk factors on the likelihood of mortality, information that could help identify the most vulnerable populations. This study was ecological in nature and there are more individual level factors that may influence COVID-19 mortality that could not be taken into account here, although an accepted and comprehensive risk factor measure from the CDC was utilized. Another limitation is the cross-sectional nature of the analysis. Future studies should investigate these relationships with mortality using a larger study period, representing a larger number of COVID-19 related deaths, to validate these findings.

## Conclusions

This analysis identifies that there are behavioral and racial/ethnic disparities in COVID-19 mortality, and highlights the most vulnerable areas in NYC that require increased focus from COVID-19 response policies. This analysis represents the first to our knowledge to investigate risk factors for COVID-19 mortality at the population-level in New York City, and supports the need for repeated and widespread testing in these communities with many risk factors for severe illness and mortality from COVID-19. Additionally, this analysis shines light on vulnerable communities that require equitable resources to rebound from COVID-19 related hardships and mortality in these communities. Future analyses could explore how the presence of comorbidities influences the immune response following COVID-19 infection, as this could ultimately be a major determinant of mortality outcomes [30]. Future vaccination efforts should understand the vulnerability to COVID-19 and mortality in NYC communities and direct attention to these areas accordingly. A successful public health response to future COVID-19 outbreaks will need to provide more attention to communities at the greatest risk in order to minimize the threat of serious illness and mortality in NYC.

## Supplementary Information

**Additional file 1: Supplementary Table 1.** Description of Risk Factor Measures. **Supplementary Table 2.** Correlations among socioeconomic status index and risk index components. Note: Spearman correlations were performed

## Data Availability

Data used in this analysis is available for open public access at https://github.com/nychealth/coronavirus-data. Individual data was not used.

## Abbreviations

ACS: American Community Survey
CDC: Centers for Disease Control and Prevention
COPD: chronic obstructive pulmonary disease
COVID-19: coronavirus disease
NYC: New York City
NYCDOH: New York City Department of Health
PCA: principal component analysis
SES: Socioeconomic status
UHF: United Hospital Fund
US: United States
ZCTA: Zip code tabulation area
