# Supplementary material for: A risk index for COVID-19 severity is associated with COVID-19 mortality in New York City
Source: BMC Public Health. 2021 Jul 24;21:1452. doi: 10.1186/s12889-021-11498-x (PMC8310407; doi:10.1186/s12889-021-11498-x)
Supplement: Supplementary file 1 — Additional file 1: Supplementary Table 1. Description of Risk Factor Measures. Supplementary Table 2. Correlations among socioeconomic status index and risk index components. Note: Spearman correlations were performed [file 12889_2021_11498_MOESM1_ESM.docx]

Supplementary Table 1- Description of Risk Factor Measures

| **CDC risk criteria** | **Proxy Variable(s) from existing NYC datasets** | **Source** | **Measure** | **Year of Data Collection** | **Prevalence in NYC (%)** |
| --- | --- | --- | --- | --- | --- |
| Diabetes | Self-reported among adults ≥18 years | 500 Cities Project | Ever told that you have diabetes? (%) | 2017 | 10.9 |
| Asthma | Self-reported, current, among adults ≥18 years | 500 Cities Project | Ever told that you have asthma? AND Do you still have asthma? (%) | 2017 | 9.7 |
| COPD, emphysema, chronic bronchitis | Self-reported among adults ≥18 years | 500 Cities Project | Ever told you have COPD, emphysema, or chronic bronchitis? (%) | 2017 | 5.6 |
| Cancer (excluding skin) | Self-reported history of cancer (excluding skin) among adults ≥18 years | 500 Cities Project | Ever told you have any other types (besides skin) of cancer? (%) | 2017 | 5.4 |
| Obese | Self-reported among adults aged ≥18 years | 500 Cities Project | BMI ≥30.0 kg/m^2^, based on self-reported height and weight (%) | 2017 | 24.4 |
| Hypertension | Self-reported among adults ≥18 years | 500 Cities Project | Ever told that you have high blood pressure? (%) | 2017 | 28.0 |
| Chronic Kidney Disease | Self-reported among adults ≥18 years | 500 Cities Project | Ever told that you have kidney disease? (%) | 2017 | 3.0 |
| Cardiovascular Disease | Self-reported coronary heart disease among adults ≥18 years | 500 Cities Project | Ever told that you have angina or coronary heart disease? | 2017 | 5.4 |
| Age 65+ years | Age | American Community Survey | Age 65+ years (%) | 2018 | 14.1 |
| Pregnancy | Birth Rate | NYC DOH Natality Data | Birth Rate (rate per 1,000) | 2015 | 14.2 |
| Previous Heart Attack | Heart attack hospitalizations among adults ≥35 years | NYC DOH Environment and Health Data | Rate (per 10,000) of heart attack hospitalizations | 2016 | 25.0 |
| Liver Disease | Hepatitis B, chronic | NYC DOH Communicable Disease Surveillance Data | Incidence rate per 100,000 | 2017 | 83.3 |
|  | Hepatitis C, chronic | NYC DOH Communicable Disease Surveillance Data | Incidence rate per 100,000 | 2017 | 59.1 |
|  | Alcohol related hospitalizations | NYS Hospital Discharge Database | Hospitalization rate (per 100,000) for alcohol morbidity or toxicity related diagnoses | 2017 | 534.7 |

Supplementary Table 2: Correlations among socioeconomic status index and risk index components

|  | Statistic | Socioeconomic Status Index |
| --- | --- | --- |
| Obesity prevalence (%) | ρ | -0.74355 |
|  | p-value | <.0001 |
| Kidney disease prevalence (%) | ρ | -0.82943 |
|  | p-value | <.0001 |
| Hypertension prevalence (%) | ρ | -0.71409 |
|  | p-value | <.0001 |
| Heart Disease prevalence (%) | ρ | -0.61804 |
|  | p-value | <.0001 |
| Diabetes prevalence (%) | ρ | -0.80396 |
|  | p-value | <.0001 |
| COPD prevalence (%) | ρ | -0.69807 |
|  | p-value | <.0001 |
| Cancer (except skin) prevalence (%) | ρ | 0.29457 |
|  | p-value | <.0001 |
| Asthma prevalence (%) | ρ | -0.71549 |
|  | p-value | <.0001 |
| Alcohol Hospitalizations Prevalence (%) | ρ | -0.24848 |
|  | p-value | 0.001 |
| Birth rate (%) | ρ | -0.17023 |
|  | p-value | 0.0256 |
| Hepatitis C prevalence (%) | ρ | -0.43988 |
|  | p-value | <.0001 |
| Hepatitis B prevalence (%) | ρ | -0.49124 |
|  | p-value | <.0001 |
| Proportion ≥ 65 years old (%) | ρ | 0.24998 |
|  | p-value | 0.0008 |

Note: Spearman correlations were performed
